# Intersection clock reveals a rejuvenation event during human embryogenesis

**DOI:** 10.1111/acel.13922

**Published:** 2023-10-02

**Authors:** Csaba Kerepesi, Vadim N. Gladyshev

**Affiliations:** ^1^ Brigham and Women's Hospital and Harvard Medical School Boston Massachusetts USA; ^2^ Institute for Computer Science and Control (SZTAKI), Eötvös Loránd Research Network Budapest Hungary

**Keywords:** aging, bisulfite sequencing, epigenetic clock, human embryogenesis, rejuvenation, RRBS, WGBS

## Abstract

Recent research revealed a rejuvenation event during early development of mice. Here, by examining epigenetic age dynamics of human embryogenesis, we tested whether a similar event exists in humans. For this purpose, we developed an epigenetic clock method, the intersection clock, that utilizes bisulfite sequencing in a way that maximizes the use of informative CpG sites with no missing clock CpG sites in test samples and applied it to human embryo development data. We observed no changes in the predicted epigenetic age between cleavage stage and blastocyst stage embryos; however, a significant decrease was observed between blastocysts and cells representing the epiblast. Additionally, by applying the intersection clock to datasets spanning pre and postimplantation, we found no significant change in the epigenetic age during preimplantation stages; however, the epigenetic age of postimplantation samples was lower compared to the preimplantation stages. We further investigated the epigenetic age of primed (representing early postimplantation) and naïve (representing preimplantation) pluripotent stem cells and observed that in all cases the epigenetic age of primed cells was significantly lower than that of naïve cells. Together, our data suggest that human embryos are rejuvenated during early embryogenesis. Hence, the rejuvenation event is conserved between the mouse and human, and it occurs around the gastrulation stage in both species. Beyond this advance, the intersection clock opens the way for other epigenetic age studies based on human bisulfite sequencing datasets as opposed to methylation arrays.

## Graphical abstract

We developed an epigenetic clock method, the intersection clock, that utilizes bisulfite sequencing in a way that maximizes the use of informative CpG sites with no missing clock CpG sites in test samples. Using the new method, we revealed a rejuvenation event during human embryogenesis around the gastrulation stage. The intersection clock opens the way for other epigenetic age studies based on human bisulfite sequencing datasets.

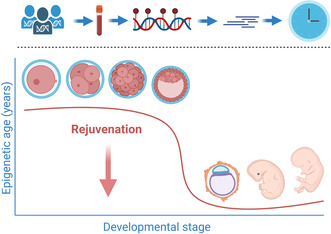

**Abbreviations**
CV: cross‐validation
DCM: dilated cardiomyopathy
DNAm: DNA methylation
ESC: embryonic stem cell
GEO: gene expression omnibus
GW: gestational weeks
HNES cells: human naïve embryonic stem cells
ICM: inner cell mass
iPSCs: induced pluripotent stem cells
MedAE: median absolute error
MDD: major depressive disorder
MII oocyte: metaphase II oocyte
P: passage
PSCs: pluripotent stem cells
RRBS: reduced representation bisulfite sequencing
SA: suicide attempters
SRA: sequence read archive
WGBS: whole genome bisulfite sequencing

## 1 INTRODUCTION

The germline as a trans‐generational cell lineage is immortal but data show it sustains age‐related changes over adult life (Jenkins et al., 2014, 2018; Lee et al., 2015). It was proposed that germline cells may be rejuvenated in the offspring after conception (Ashapkin et al., 2017; Gladyshev, 2020). Recently, we carried out a data‐driven test of this idea, wherein epigenetic clocks were applied to track changes in biological age (i.e., the age based on molecular markers) and revealed a rejuvenation event during the early stages of mouse embryogenesis (Kerepesi et al., 2021). We found that the mean epigenetic age of embryonic day 6.5/7.5 samples was consistently lower compared to the earlier stages of embryogenesis, and single‐cell analyses using a different, probabilistic clock (scAge), showed a similar pattern (Trapp et al., 2021). This embryonic period corresponds approximately to gastrulation. However, it remained an open question whether a similar rejuvenation event exists in humans. To answer this question, here we evaluated the epigenetic age dynamics of human early embryogenesis.

Epigenetic clocks, based on methylation levels of CpG sites, emerged as a promising molecular estimator of biological age (Bell et al., 2019; Fabris et al., 2017; Galkin et al., 2020; Horvath & Raj, 2018). These clocks were shown to quantitatively measure numerous aspects of human aging (Breitling et al., 2016; Hannum et al., 2013; Horvath, 2013; Horvath et al., 2015; Horvath & Ritz, 2015; Levine et al., 2018; Lin et al., 2016; Lu et al., 2019; Maierhofer et al., 2017; Marioni, Shah, McRae, Chen, et al., 2015; Marioni, Shah, McRae, Ritchie, et al., 2015; Weidner et al., 2014). Although multiple epigenetic clocks are available based on human methylation arrays, there has been no available aging clock based on human bisulfite sequencing. It may be because, until recently, there were no available bisulfite sequencing data (with a sufficient number of samples with age metadata) suitable for training an epigenetic clock.

## 2 RESULTS

**FIGURE 1:**
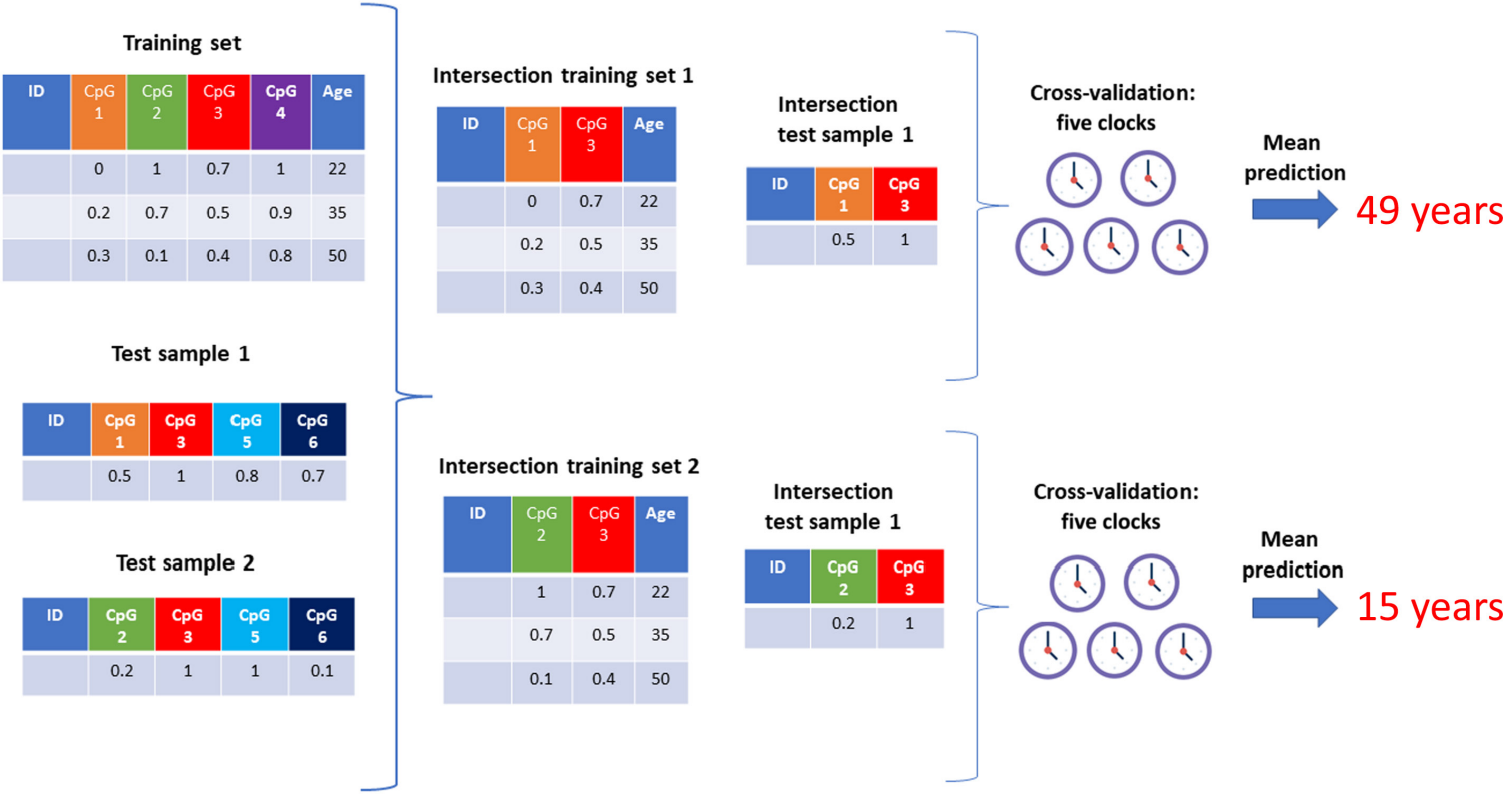
Schematics of the intersection clock workflow. For each test sample, we perform the following workflow separately: first, we determine the intersection of CpG sites between the training data set and the test sample. Then, we restrict the training set and the test sample to the intersected CpG sites and use the restricted training set (*intersection training set*) and test sample (*intersection test sample*) for training and testing. We perform a fivefold cross‐validation on the intersection training set that results in five intersection clocks (e.g., ElasticNet models). Finally, the mean prediction of the five clocks yields the predicted (epigenetic) age of the sample.

**FIGURE 2:**
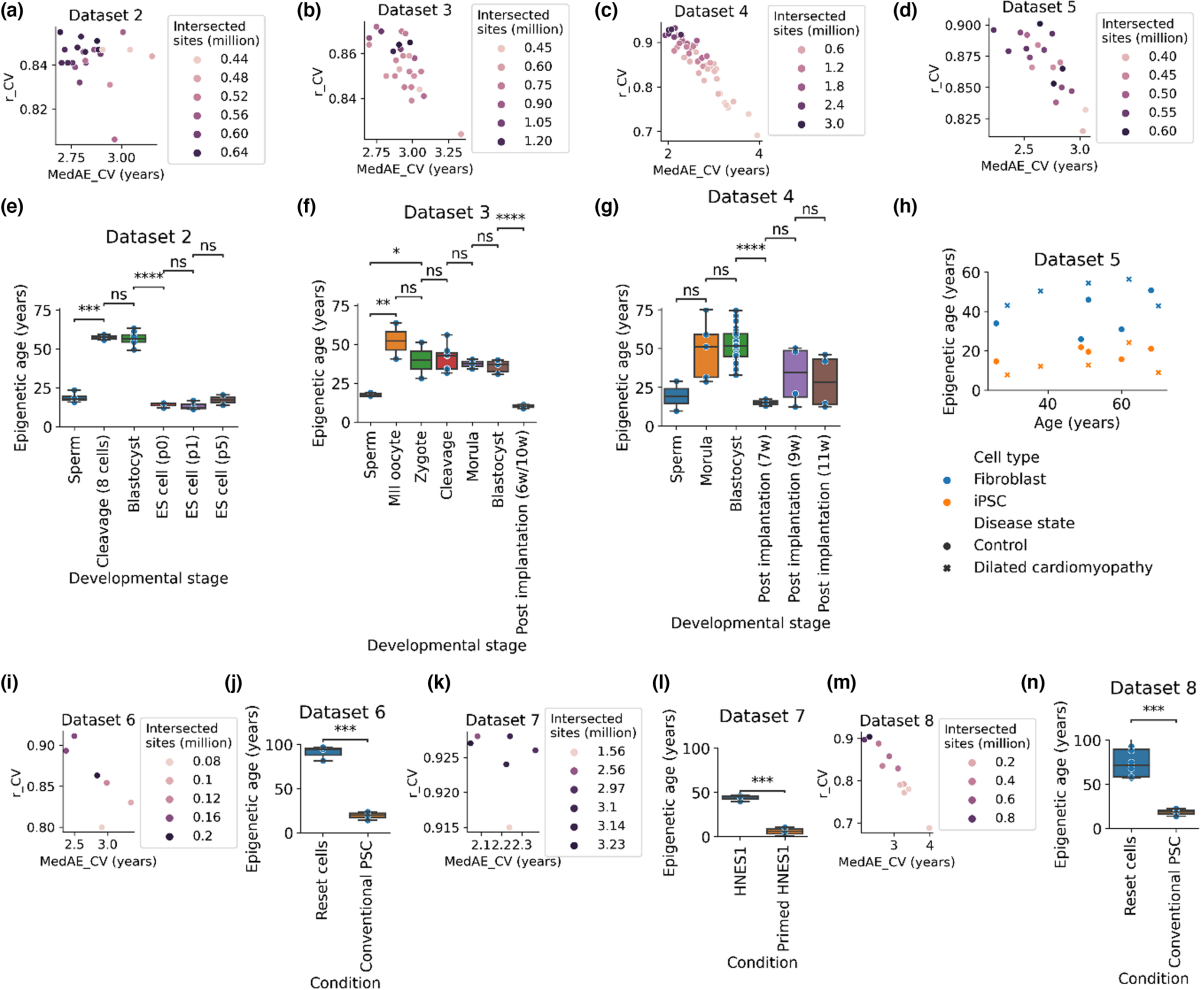
Intersection clock reveals a rejuvenation event during early human embryogenesis. (a–d, i, k, m) Number of intersected CpG sites and cross‐validation (CV) model performance, assessed by Pearson correlation coefficient (*r*) and median absolute error (MedAE), of the intersection clock for Datasets 2–8, respectively. Each dot represents a test sample and the CV performance was measured on the training set (Dataset 1) restricted to the intersected CpG sites. (e–g) Mean predicted age (epigenetic age) of samples from different human developmental stages assessed by the intersection clock for Datasets 2–4. ES cells of Dataset 2 represent postimplantation epiblast. (h) Mean predicted age (epigenetic age) of fibroblasts and derived iPSCs of control and dilated cardiomyopathy patients assessed by the intersection clock for Dataset 5. (j, l, n) Mean predicted age (epigenetic age) of samples from naïve (reset cells, HNES1 cells) and primed (conventional PSCs, primed HNES1 cells, representing postimplantation epiblast) ESC cells assessed by the intersection clock for Datasets 6–8.

To evaluate the epigenetic age dynamics of human embryogenesis, we needed an epigenetic clock method based on human bisulfite sequencing data, as only this type of data are available for human embryos (Table S1). Thus, we developed a new epigenetic clock method, the “intersection clock” (Figure 1) and trained it on available reduced‐representation bisulfite sequencing (RRBS) data of 182 human blood samples (Dataset 1 in Table S1, Figure S1) (Bhak et al., 2019). The intersection clock is a novel concept optimized for the use of bisulfite sequencing data where there is an insufficient number of overlapped CpG sites between the training and testing data sets for reliable predictions. The idea behind the intersection clock is to maximize the use of informative CpG sites with no missing clock CpG sites in test samples. For this purpose, it predicts the age of a test sample by training and testing on the intersected CpG sites between the training dataset and the test sample data. So, the intersection clock method guarantees to have no missing clock CpG sites in test samples. Although such an approach requires training new epigenetic clocks for each dataset, it maximizes the utility of these datasets. To evaluate epigenetic age dynamics during embryogenesis, we collected available human DNA methylation (DNAm) datasets (Dataset 2–4) (Guo et al., 2014; Smith et al., 2014; Zhu et al., 2018) and analysed them with the intersection clock. The intersection clock showed high performance as assessed by cross‐validation on the training set restricted to the intersected CpG sites (Figure 2a–c). Dataset 2 is from a study that generated genome‐scale methylation maps of human preimplantation development and embryonic stem cells (representing human epiblast) using RRBS. We processed methylation levels for all human samples including sperm, cleavage, blastocysts, and human embryonic stem (ES) cells. We observed no significant change in the predicted epigenetic age between cleavage stage‐ and blastocyst stage embryos (*p* = 0.7378); however, there was a significant decrease in epigenetic age between blastocysts and ES cells (*p* = 1.582e−12) (Figure 2e). A cross‐species comparison between mouse epiblast (E6.5) and human ES cells suggests that these ES cells are a reasonable proxy for human epiblast (Smith et al., 2014); therefore our data suggest that the epigenetic age of human postimplantation epiblasts is also lower than the epigenetic age of preimplantation stages. We further applied the intersection clock to two other datasets (Datasets 3 and 4) containing preimplantation (zygote, cleavage, morula, and blastocyst) and postimplantation (GW 6–11 liver and villus) samples. We observed no significant change among preimplantation stages; however, there was a significant decrease in the epigenetic age of postimplantation samples compared to preimplantation stages (Figure 2f,g). Together, our data suggest that human embryos are rejuvenated during early embryogenesis.

As a further validation of the capability of our method to measure rejuvenation, we applied the intersection clock to a human iPSC dataset (Dataset 5) (Morival et al., 2021). We observed a significant decrease in epigenetic age after reprogramming fibroblasts from adult control (*p* = 0.0136) and dilated cardiomyopathy patients (2.679e−05) (Figure 2d,h).

Datasets 2–4 do not only contain embryo data, but also sperm data, and in Dataset 3, MII oocytes samples. Applying the intersection clock to the methylation profiles of these samples, we found that the epigenetic age of sperm cells was in some cases lower than that of preimplantation stages: the difference was significant in the case of Datasets 2 and 3 (Figure 2e,f), and not significant in the case of Dataset 4 (Figure 2g). On the other hand, we observed no significant difference in the epigenetic age between human MII oocytes and preimplantation embryos (Figure 2f), although the epigenetic age of sperm cells was significantly lower compared to MII oocytes. It is unknown whether male germ cells may also undergo rejuvenation during gametogenesis.

Conventional human pluripotent stem cells (PSCs), whether derived from blastocysts or generated by reprogramming, differ from mouse embryonic stem cells and are considered to represent a developmentally advanced, or primed, stage of pluripotency (Guo et al., 2016). Multiple methods were developed for the generation of a more naïve‐like phenotype by in vitro resetting of conventional PSCs (Guo et al., 2017; Takashima et al., 2014) or direct cell capturing from the ICM (Guo et al., 2016). Here, we investigated the epigenetic age of primed (representing early postimplantation) and naïve (representing preimplantation) PSCs by applying the intersection clock to Datasets 6–8 and observed that in all cases the epigenetic age of primed cells was significantly lower than that of naïve cells (Figure 2i–n).

Altogether, these data support the hypothesis that the rejuvenation event occurs during early embryonic development in human.

## 3 DISCUSSION

Our results recapitulate the findings in mice (Kerepesi et al., 2021) where we found that the epigenetic age of embryonic day 6.5/7.5 samples was consistently lower compared to that of earlier stages of embryogenesis. Thus, the embryonic rejuvenation event is conserved between the mouse and human and it occurs around the gastrulation stage in both species. Our data support the model that ground zero, corresponding to the lowest biological age of an organism, is achieved during human embryogenesis (Gladyshev, 2020). As our knowledge about ground zero is developing, it may change our thinking about the beginning of human organismal life.

We found that the epigenetic age of sperm cells was in some cases lower than that of MII oocytes and preimplantation stage embryo. Our study examined embryonic rejuvenation, and it is unknown whether germ cell development and gametogenesis are also associated with rejuvenation. Further studies are needed to clarify this point.

In the only available potential training dataset (Dataset 1), the oldest healthy patient was 40 years old, whereas older patients exhibited adverse health phenotypes (SA or MDD, Table S2, Figure S1). Although the original study revealed significant differences in the methylation profiles of healthy vs SA and healthy versus MDD groups, it may be that the difference, at least in part, is due to the average age differences between the healthy group and the SD/MDD group. It is also possible that the CpG sites that differentiate between healthy and SD/MDD groups are not the same as those used by epigenetic clocks. Altogether, we think, in this case, it is reasonable to use all data for the training process.

Previous epigenetic age assessment of human iPSCs based on methylation arrays showed a decline in epigenetic age (i.e., rejuvenation) during reprogramming (Horvath, 2013; Olova et al., 2019). We confirmed these results based on human bisulfite sequencing data (Figure 2h) and showed that despite the issues discussed above regarding the training dataset, the intersection clock can detect rejuvenation.

Global CpG methylation of the human embryo decreases during preimplantation development, from the zygote to blastocyst stages followed by an increase of global CpG methylation in the postimplantation embryo (represented by primed PSCs) (Guo et al., 2014, 2016, 2017; Smith et al., 2014; Takashima et al., 2014; Zhu et al., 2018). In contrast, we observed no significant changes in epigenetic age during preimplantation development (zygote, cleavage, morula and blastocyst stages; Figure 2e–g) followed by a decrease of epigenetic age in the postimplantation embryo (represented by primed PSCs) (Figure 2e–g,j,l,n). These data suggest that DNA methylation maintenance and de novo methylation have roles in the rejuvenation event as observed previously in mice (Kerepesi et al., 2021; Trapp et al., 2021).

## 4 EXPERIMENTAL PROCEDURES

### 4.1 Collection of early human embryos and gametes

Our study did not involve experimentation with human embryos or embryonic samples. Instead, we relied on publicly available deidentified data and computational tools. However, we described the relevant information in the Supplementary Information (Data S1).

### 4.2 Training data set

We downloaded bisulfite‐sequencing reads of Dataset 1 from the Sequence Read Archive (SRA) database (PRJNA531784) by using the prefetch program of the sratoolkit (v2.10.4). This process resulted in a paired‐end read file (SRR format) for each of the 182 peripheral blood samples. The samples were collected from 56 suicide attempters (SAs), 39 patients with major depressive disorder (MDD), and 87 healthy controls (Figure S1, Table S2). Then, we used the fastqdump program of the sratoolkit (v2.8.2) for extracting the fastq files. Reads were trimmed and quality filtered by TrimGalore! v0.6.4 using the –rrbs option for RRBS. Methylation levels were extracted using Bismark v0.22.2 with Bowtie 2 mapping to the GRCh38 human genome assembly. Using the CpG report output files of Bismark, we summarized the methylated and unmethylated reads for both strands (negative and positive) and calculated the methylation percentages of each strand by using the summarized values. In the final feature table, we considered only the CpG sites that are covered by at least five reads for all 182 training samples.

### 4.3 Test data sets

We downloaded processed methylation data from Gene Expression Omnibus (GEO) and selected human bulk samples for our analysis (Datasets 2–8, Table S1). We used the Python package liftover for lifting hg19 (GRCh37) genomic positions to hg38 (GRCh38) for every dataset. In the case of Datasets 2 and 6 we first shifted the 0‐based genomic coordinates by 1 before lifting. We considered only the CpG sites that are covered by at least five reads.

### 4.4 The intersection clock workflow

The idea behind the intersection clock is to maximize the use of informative CpG sites in the training and test sets. For each test sample, we performed the following workflow separately: first, we determined the intersection of CpG sites between training and test datasets. Then, we restricted the training set and the test sample to the intersected CpG sites. Subsequently, we used the restricted training set and test sample to train and test a clock as follows: we trained an ElasticNet regression model using random 80% of the samples of the restricted training set and validated on the remaining 20% of samples. We optimized the lambda parameter by the built‐in 10‐fold cross‐validation of the Python package Glmnet (https://github.com/civisanalytics/python‐glmnet, v2.2.1; alpha = 0.5, n_splits = 10). The final model was used to predict the age of the restricted test sample. To achieve a more robust prediction, we performed a fivefold cross‐validation (CV) repeating the same procedure as above. The fivefold CV resulted in five clocks for each test sample. In this study, we used 182 human blood samples from Dataset 1 as a training set and Datasets 2–8 for testing. We considered only the test samples that had at least 10,000 intersected CpG sites.

### 4.5 Statistical analysis

We used the Python packages SciPy (v1.3.1) and Scikit‐learn (v0.23.2) for statistical analysis. Two‐sided t‐tests were calculated for comparing two groups: ns, *p* > 0.05; *, 1 × 10^−2^ < *p* ≤ 5 × 10^−2^; **, 1 × 10^−3^ < *p* ≤ 1 × 10^−2^; ***, 1 × 10^−4^ < *p* ≤ 1 × 10^−3^; ****p ≤ 1 × 10^−4^. In the analysis of fibroblasts and derived iPSCs (Figure 2h), we used paired *t*‐tests. Correlations were evaluated by Pearson correlation coefficient (*r*).

## AUTHOR CONTRIBUTIONS

CK and VNG conceived the study. CK acquired data, performed data analysis, and developed the intersection clock. VNG supervised the study. CK and VNG wrote the manuscript.

## CONFLICT OF INTEREST STATEMENT

The authors have no conflict of interest to declare.

## Supporting information


Data S1

Table S2

Table S3

## Data Availability

Python codes of the intersection clock workflow and output files for this study (e.g., predicted values and clock models of the CVs) are available on GitHub: https://github.com/kerepesi/IntersectionClock. Prediction tables of the intersection clock that were used for the generation of Figure 2 are available in Table S3.
