# Supplementary material for: Intersection clock reveals a rejuvenation event during human embryogenesis
Source: Aging Cell. 2023 Oct 2;22(10):e13922. doi: 10.1111/acel.13922 (PMC10577537; doi:10.1111/acel.13922)
Supplement: Supplementary file 1 — Data S1 [file ACEL-22-e13922-s001.docx]

**SUPPLEMENTARY INFORMATION AND FIGURES**

**Collection of early human embryos and gametes:** Our study did not involve experimentation with human embryos or embryonic samples. Instead, we relied on publicly available deidentified data and computational tools. However, we describe here relevant information about the collection of embryos and gametes (including donor age when available):

- Dataset 2 (Smith et al., 2014): Couples donating surplus embryos, resulting from infertility treatment, for the purpose of research signed an extensive consent form at the time of their donation. These consent forms were approved by the Harvard University IRB. Semen samples were collected from five healthy patients between the ages of 30 and 34 undergoing an evaluation for infertility. Each male was a non-smoker with a body mass index < 30 kg/m^2^.
- Dataset 3 (H. Guo et al., 2014): This study was approved by the Reproductive Study Ethics Committee of Peking University Third Hospital. All of the gametes and embryos were collected voluntarily after obtaining written informed consents signed by donor couples. The average age of donors in this study was 30 years old. Oocytes, sperm, zygotes, 2-cell stage and 4-cell stage embryos were voluntarily donated by healthy volunteers who have already had one or two healthy children from a natural pregnancy. Oocytes were obtained from the donors at the Center for Reproductive Medicine in Peking University Third Hospital using standard clinical protocols. Embryos, at the 8-cell, morula and blastocyst stages were donated by couples who had undergone in vitro fertilization (IVF) treatments. These donor couples, whose infertility is purely due to female tubal factors, had a healthy baby through the IVF cycle already.
- Dataset 4: All human gametes and early embryos were obtained from donors voluntarily at the Center for Reproductive Medicine of Peking University Third Hospital after signing informed consent and following the approval of the Ethics Committee. The human semen sample used in this study was obtained from one healthy man with normal semen parameters.
- Dataset 6: Human embryo research was licensed by the UK Human Fertilization and

Embryology Authority. Supernumerary embryos donated from in vitro fertilization programs with informed consent were thawed and cultured to day 7 post fertilization

- Dataset 7: Supernumerary frozen human embryos were donated with informed consent under license from the UK HFEA.

**SUPPLEMENTARY TABLE S1** Embryonic DNA methylation datasets and samples used in this study. We selected human RRBS/WGBS bulk samples from available datasets. DCM, dilated cardiomyopathy; ES cell, embryonic stem cells; GW, gestational weeks; HNES cells, human naïve embryonic stem cells; iPSCs, induced pluripotent stem cells; MII oocyte, metaphase II oocyte; P, passage; PSCs, pluripotent stem cells; RRBS, reduced representation bisulfite sequencing; WGBS, whole genome bisulfite sequencing.

| **Name / Ref** | **Reference** | **Accession** | **Platform** | **Species** | **Samples (number of biological replicates)** |
| --- | --- | --- | --- | --- | --- |
| Dataset 1 | (Bhak et al., 2019) | PRJNA531784 | RRBS | Human | Blood samples from various ages |
| Dataset 2 | (Smith et al., 2014) | GSE51239 | RRBS | Human | Sperm (4), cleavage (2), blastocyst (9), ES cells P0-P5 (8) |
| Dataset 3 | (H. Guo et al., 2014) | GSE49828 | RRBS | Human | Sperm (4), MII oocyte (2), zygote (2), cleavage (7), morula (3), blastocyst (6), post-implantation GW 6 (2), post-implantation GW 10 liver (1) |
| Dataset 4 | (Zhu et al., 2018) | GSE81233 | WGBS | Human | Sperm (2), morula (5), blastocyst (35), post-implantation GW 7 to GW 11 villus (4) and heart (6) |
| Dataset 5 | (Morival et al., 2021) | GSE164365 | RRBS | Human | Fibroblast and their iPSC derivatives from DCM patients (5, 5) and control (5, 5) |
| Dataset 6 | (Takashima et al., 2014) | GSE60945 | WGBS | Human | Conventional PSCs (3), reset cells (3) |
| Dataset 7 | (G. Guo et al., 2016) | E-MTAB-4462 | WGBS | Human | HNES1 cells (3), primed HNES 1 cells (3) |
| Dataset 8 | (G. Guo et al., 2017) | GSE90168 | WGBS | Human | Conventional PSCs (3), reset cells (8) |

**
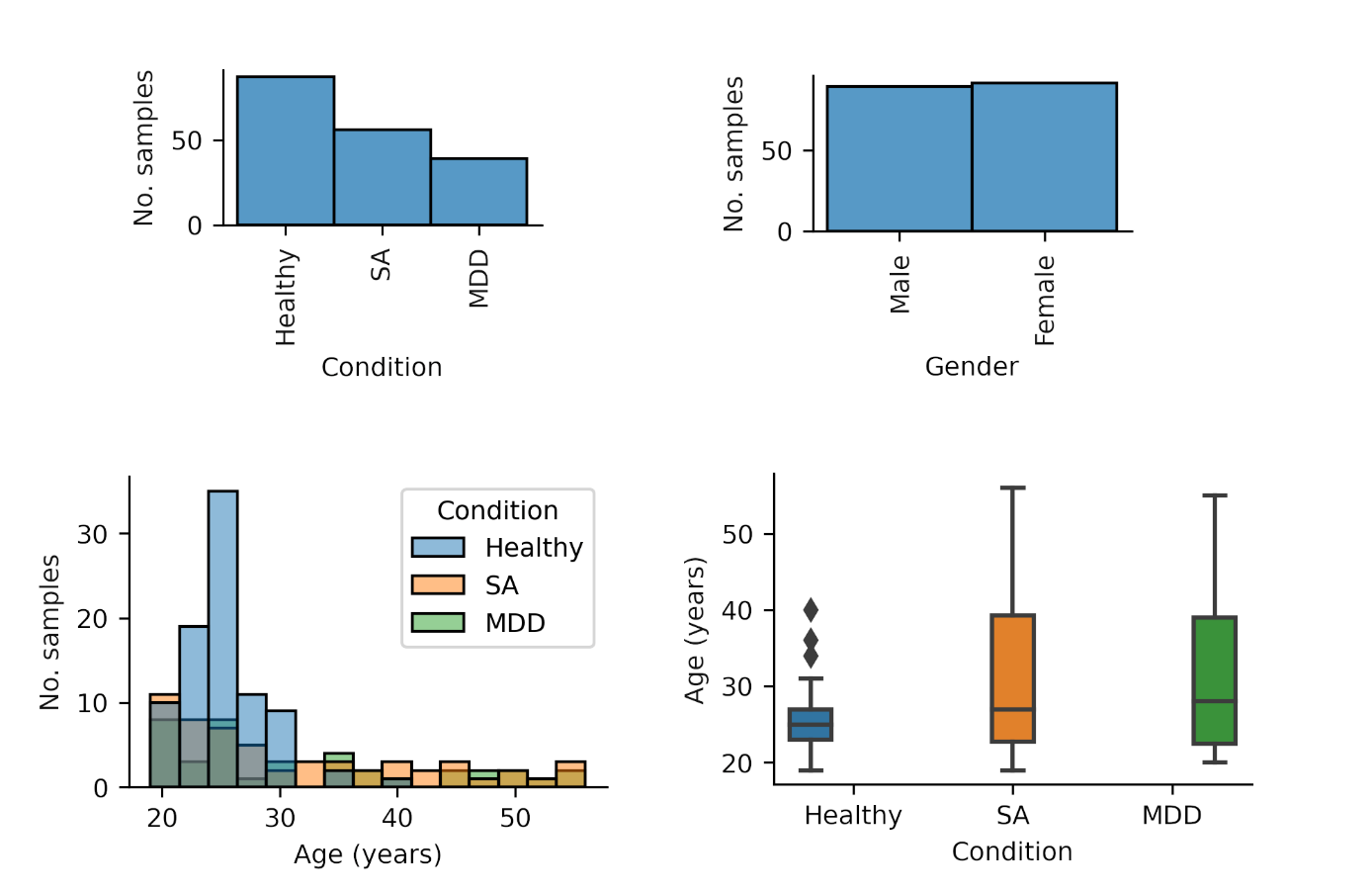
**

**SUPPLEMENTARY FIGURE S1** Distribution of 182 training samples (human blood) by condition, gender and age. SA, suicide attempters; MDD, major depressive disorder.
